# Mucin Coatings Establish Multifunctional Properties on Commercial Sutures

**DOI:** 10.1021/acsabm.4c01793

**Published:** 2025-02-27

**Authors:** Ufuk Gürer, Di Fan, Zhiyan Xu, Qaisar Nawaz, Jorrit Baartman, Aldo R. Boccaccini, Oliver Lieleg

**Affiliations:** 1 Department of Materials Engineering, School of Engineering and Design, Technical University of Munich, Boltzmannstraße 15, Garching 85748, Germany; 2 Center for Protein Assemblies (CPA), Munich Institute of Biomedical Engineering (MIBE), Technical University of Munich, Ernst-Otto-Fischer Straße 8, Garching 85748, Germany; 3 Institute of Biomaterials, Department of Materials Science and Engineering, University of Erlangen-Nuremberg, Cauerstraße 6, Erlangen 91058, Germany

**Keywords:** biopolymer, antibacterial, surgical site infection, bioactive glass, drug release

## Abstract

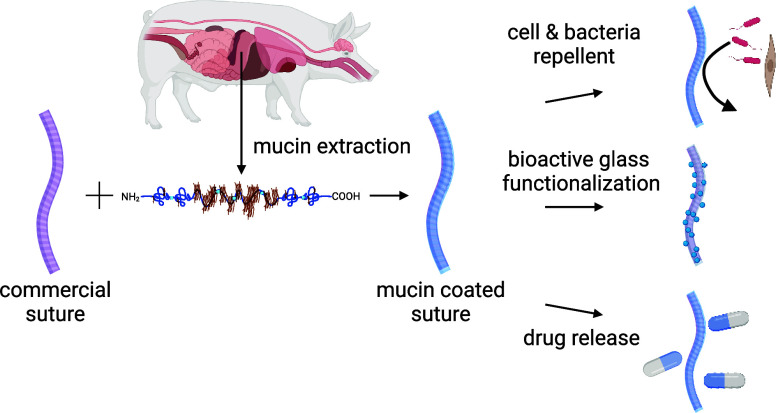

During the wound healing process, complications such as bacterial attachment or inflammation may occur, potentially leading to surgical site infections. To reduce this risk, many commercial sutures contain biocides such as triclosan; however, this chemical has been linked to toxicity and contributes to the occurrence of bacterial resistance. In response to the need for more biocompatible alternatives, we here present an approach inspired by the innate human defense system: utilizing mucin glycoproteins derived from porcine mucus to create more cytocompatible suture coatings with antibiofouling properties. By attaching manually purified mucin to commercially available sutures through a simple and rapid coating process, we obtain sutures with cell-repellent and antibacterial properties toward Gram-positive bacteria. Importantly, our approach preserves the very good mechanical and tribological properties of the sutures while offering options for further modifications: the mucin matrix can either be condensed for controlled localized drug release or covalently functionalized with inorganic nanoparticles for hard tissue applications, which allows for tailoring a commercial suture for specific biomedical use cases.

## Introduction

Throughout history, supporting the wound healing process has been a challenge. Improperly treated lesions are prone to bacterial infection and inflammation, which prevents healing and leads to long-lasting wounds.^1−3^ Although using antibacterial sutures for closing a wound can help,^4^ sutures can still be a trigger for inflammation^5,6^ and serve as a target for bacterial colonization and biofilm formation, and those events are main causes of SSIs.^7−9^ Such SSIs can further develop into organ or space infections^10,11^ and, in severe cases, even lead to sepsis.^12,13^ To mitigate these risks, a range of antibacterial sutures has been developed; however, only a few have proven to be effective.

One example for such an efficient antibacterial suture (Vicryl Plus, developed by Johnson & Johnson) carries a coating of the chemical 5-chloro-2-(2,4-dichlorophenoxy) (triclosan). Whereas triclosan serves as a primary antiseptic and disinfectant in clinical settings, where it has proven to be effective in reducing SSIs,^14,15^ there are concerns that a prolonged exposure to triclosan may impact the health of patients.^16^ For instance, it has been shown that high levels of triclosan may alter the gene expression through interactions with the pregnane X receptor and the constitutive androstane receptor, both of which are involved in regulating endocrine functions.^17,18^ Additionally, excessive use of triclosan has been associated with liver toxicity and tumor growth as demonstrated in *in vivo* studies.^19−21^ For instance, research by Yang et al.^22^ suggests that triclosan exposure can lead to low-grade colonic inflammation and exacerbate colitis symptoms in mice, thus potentially increasing the risk of colitis-associated colon cancer. In addition, some people are allergic to triclosan,^23^ and the widespread use of triclosan raises environmental concerns, as it accumulates in marine ecosystems, impacts aquatic life^24,25^ and contributes to the global health risk of microbial resistance.^26^

These findings explain the growing interest in developing alternatives to triclosan-coated sutures that provide effective antibacterial properties while minimizing health and environmental risks. In response to this need, researchers have explored various innovative materials and coatings for sutures.^27,28^ For instance, Ghosh et al.^29^ created sutures with dual antibacterial and anti-inflammatory properties by incorporating *inter alia* quaternary benzophenone-based agents onto the suture base material. Zhang et al.^30^ employed the organic polymer polypyrrole as an antibacterial suture coating, and Wang et al.^31^ produced silk fibroin-based sutures containing berberine and artemisinin, with these additives providing antimicrobial and anti-inflammatory properties to the biocompatible silk matrix,^32^ respectively. However, despite these promising developments, many of these formulations still rely heavily on chemical compounds that are not fully biocompatible.^33 −35^ In addition to these chemical modifications, surface treatments such as plasma etching^36^ and nanostructured surfaces^37^ have been explored to develop bacteria-repellent surfaces. However, these methods often only provide short-term effects and may weaken the mechanical properties of the treated sutures and/or increase the friction behavior of the sutures when sliding through tissue.

Instead of incorporating potentially cytotoxic chemicals to sutures to obtain antibacterial properties, one could also develop sutures from a biomaterial with intrinsically high biocompatibility and antibacterial/anti-inflammatory properties. Tissue decellularization can be used to create such biomaterials and results in extracellular matrix-rich scaffolds that offer excellent biocompatibility and reduced immune response, thus making them promising candidates for wound regeneration applications.^38,39^ Recently, Lee et al.^40^ introduced a suture made from decellularized porcine gut, which naturally possesses anti-inflammatory properties. Whereas this innovative approach shows promise, the decellularization process is labor-intensive and costly, which limits its practicality for widespread clinical use compared to conventional sutures and/or coating approaches.

Here, we make use of a coating strategy but employ a biopolymer, *i.e*., the lab-purified porcine gastric mucin (MUC5AC), to create coatings on commercially available Vicryl sutures. Mucins are the key biopolymeric component in the mucosal systems covering all wet epithelia and offer excellent biocompatibility, superior tribological performance, and antibiofouling properties.^41,42^ Such highly functional mucins were manually purified and covalently attached to the surfaces of two types of Vicryl sutures, one of which carries a triclosan coating. The generated mucin coating is not only more cytocompatible than the triclosan-coated variant but also effectively prevents cell attachment, which otherwise can be a key contributor to inflammation. Additionally, it exhibits antibiofouling properties, especially against Gram-positive bacteria, but maintains the mechanical stability of the sutures and their very good sliding properties. Moreover, the mucin layer allows for loading therapeutic drugs onto the suture surface and enables their triggered release. We can further modify the mucin coated sutures through the covalent attachment of bioactive glass nanoparticles, thus expanding their potential use for applications in hard tissue repair (*e.g.*, for dental applications). This innovative approach renders our mucin-coated sutures a versatile and adjustable platform for surgical applications, offering improved safety, efficacy, and adaptability.

## Materials and Methods

If not stated otherwise, all chemicals were purchased from Carl Roth GmbH (Karlsruhe, Germany).

### Mucin Purification

Mucin purification was conducted as described in Marczynski et al.^43^ In brief, raw mucus was manually scraped from the inner tissue of fresh porcine stomachs obtained from a local butcher. The collected mucus was first diluted 5-fold in a phosphate-buffered saline (PBS, 10 mM, pH 7.0) solution containing 170 mM sodium chloride (NaCl) and 0.04% (w/v) of the bactericide sodium azide, and then stirred at 4.0 °C overnight. Subsequently, size exclusion chromatography was conducted using an ÄKTA purifier system (GE Healthcare, Chicago, IL, USA) and an XK50/100 column packed with Sepharose 6FF (GE Healthcare) to separate the mucins from smaller molecules. The resulting solutions were pooled, dialyzed against ultrapure water (UPW), and concentrated by cross-flow filtration using an ultrafiltration hollow fiber cartridge (MWCO = 100 kDa, UFP-100-E-3MA, GE Healthcare). After lyophilization, the mucin was stored at −70 °C until further use.

### Applying a Covalent Mucin Coating to Commercial Sutures

Vicryl (3–0) and Vicryl Plus (3–0) sutures, each approximately 45 cm in length, were purchased from Ethicon (Johnson & Johnson, New Brunswick, NJ, USA). The as received sutures are already coated with a lubricating layer of calcium stearate.^44^ In addition, the Vicryl Plus variant contains the antibacterial molecule triclosan.^45^ To covalently attach MUC5AC to the sutures, carbodiimide chemistry was employed.^46^ Briefly, the sutures were incubated for 1 h in 15 mL 2-(N-morpholino)ethanesulfonic acid buffer (MES, 100 mM, pH 5.0) containing 20 mg 1-ethyl-3-(3dimethylaminopropyl) carbodiimide hydrochloride (EDC) and 20 mg *N*-hydroxysulfosuccinimide sodium salt (NHS, 98%, abcr GmbH, Karlsruhe, Germany). Following this step, the sutures were placed in a PBS solution (pH = 7.4) containing 0.15% (w/v) manually purified mucin and incubated overnight at 4 °C. Afterward, the samples were rinsed with 80% ethanol and distilled water (D.I water), then further washed with 80% ethanol for 30 min to remove physically bound mucin macromolecules, and finally left to dry at room temperature under a fume hood.

### Verification of the Mucin Coating

To verify the successful application of the mucin coating, fluorescently labeled mucin was used to coat the sutures. First, a fluorescent dye (ATTO 594, carboxy modified, ATTO-TEC GmbH, Siegen, Germany, excitation/emission = 603/626 nm) was covalently attached to the mucin via carbodiimide coupling. Briefly, a stock solution (10.0 mg mL^–1^) of the dye was prepared in D.I water. Then, the solution was diluted to a concentration of 0.33 mg mL^–1^ in 1 mL of MES buffer (10 mM, pH 5.0). Next, 20 mg of EDC and 20 mg of NHS were added to the solution and incubated at room temperature for 3 h. This mixture was added to a mucin solution (40 mg MUC5AC in 19 mL of 10 mM PBS, pH 7.0) and incubated in the dark at room temperature for 3 h. To remove unbound dye and reactants, the solution was dialyzed against D.I water for 2 days using a semipermeable membrane (300 kDa, Spectrum, Spectra/Por, Spectrum Laboratories, Inc., USA). During this dialysis step, the D.I water was exchanged three times. Afterward, the functionalized MUC5AC was freeze-dried and stored at −70 °C until further use. Following a carbodiimide-based coupling of fluorescently labeled mucin to the suture surface (following the protocol described above), microscopy images were acquired using a Leica DMi8 microscope (Leica, Wetzlar, Germany) equipped with a 10X lens (N PLAN 10*X*/0.25 DRY) and a digital camera (Orca Flash 4.0 C11440, Hamamatsu, Japan). The surface structure of both, the as-received and (fluorescently labeled) mucin-coated sutures was detected using a Texas Red filter set (TXR, ex. = 540–580 nm, DC = 585 nm, em. = 592–668 nm, Leica). The exposure time was set to 100 ms without employing binning.

### Tensile and Ex Vivo Friction Tests

Tensile and friction tests were conducted using a lab-built pulling device, which is described in detail in Naranjo et al.^47^ For each test, the sutures were cut into pieces of ∼15 cm length. One end of the suture was secured to a screw with a double knot, while the other end was turned into a loop, which was attached to a hook of the pulling device. The suture pieces were then stretched at a speed of ∼15 mm s^–1^ until they ruptured. During this test, the device recorded a force–distance curve, from which the maximum rupture force was determined.

Friction tests were carried out on freshly cut porcine skin and chicken stomach samples. The porcine skin samples were fixed to a sample holder, and a suture was threaded through a needle that was used to penetrate the skin. Then, a loop was created at the end of the suture and attached to the hook of the pulling device; the suture was finally pulled through the skin over a distance of ∼9 cm at a speed of ∼15 mm s^–1^. For friction tests conducted with the chicken stomach samples, some adjustments were made to this protocol to increase the load on the sutures during sliding while guaranteeing good reproducibility and minimizing the impact of sample-to-sample variations. Initially, a suture was pulled several times through the same stomach location to monitor (putative) changes in the friction response. Having confirmed that the tissue sample could withstand such consecutive pulling events without tissue disintegration or systematic changes to the obtained friction response, one-half of a set of suture samples was partially coated with mucin, whereas the other half remained uncoated. These semicoated sutures were then pulled through chicken stomach samples (using the same stomach sample for a given suture) over a distance of ∼9 cm pulled per test; this protocol allowed for conducting up to four consecutive measurements per suture sample (which has a total length of 45 cm). After recording the corresponding force–distance curves, friction energies were calculated by integrating the area under the force–distance curve.

### Tissue Damage Tests

To investigate suture-induced wear formation on tissue, chicken stomach samples were selected as they were already used in a subset of the friction tests. First, a chicken stomach was cut open and placed into a sample holder. Then, a hook was attached to a beam, onto which one end of the suture (45 cm in length) was fixed by a knot. A small counterbalance weight of 2.8 g was connected to the other end of the suture to ensure consistent contact between the suture and the stomach tissue. The same pulling device employed for the friction tests was used to drag the suture across the freshly cut tissue at a constant pulling velocity of 15 mm s^–1^. This movement was repeated exactly 10 times along the same path. To minimize the impact of sample-to-sample variations, the same tissue piece was used for a sliding process conducted with both, a mucin-coated and an uncoated suture. After this sliding process, profilometric images of the damaged tissue areas were captured using a laser scanning microscope (VK-X1000 series, Keyence Corporation, Osaka, Japan) equipped with a 5X lens. A minimum of two square images (each having an area of 1000 μm^2^) were captured for each sample. Then, the acquired topographical data was analyzed and processed using the software MultiFileAnalyzer (Keyence Corporation) to correct for distortions caused by sample tilt. After this image processing step, the metrological surface parameter developed interfacial area ratio (*S*_dr_) was calculated from the corrected images.

### Cell Culture

Human epithelial cells (HeLa) were cultivated in Minimum Essential Medium Eagle (MEM, Sigma-Aldrich) supplemented with 10.0% (v/v) fetal bovine serum (FBS; Sigma- Aldrich), 2 × 10^–3^ M l-glutamine solution (Sigma-Aldrich), 1.0% (v/v) nonessential amino acid solution (NEAA; Sigma-Aldrich), and 1.0% penicillin/streptomycin (Sigma-Aldrich). Mouse fibroblasts (NIH/3T3) were cultured in Dulbecco’s Eagle’s high glucose Medium (DMEM, Sigma-Aldrich) supplemented with 10.0% fetal bovine serum (FBS) and 1.0% penicillin/streptomycin. Both cell lines were incubated at 37 °C and 5% CO_2_ in a humidified atmosphere.

### Cytotoxicity Test

A human epithelial cell line (HeLa) was used as a model system for those cytotoxicity tests. Putative cytotoxic effects of the different sutures were evaluated according to ISO 10993–5. In detail, a ∼ 11 cm piece of each suture variant was immersed in 1.5 mL of the complete culture medium for 24 and 48 h, respectively, to allow chemical compounds from the suture to leach out; fresh medium (which was not subjected to an incubation step with a suture sample) was used for the control groups. Subsequently, a WST-1 (water-soluble tetrazolium 1) assay was employed to assess the cytotoxicity of this “leaching” medium. For this step, HeLa cells were seeded into the wells of 96-well plates (5000 cells/well) and incubated for 24 h at 37 °C. Afterward, the cell culture medium was replaced with a sterile filtered “leaching” medium. After 24 h of incubation, the leaching medium was replaced with a 2.0% (v/v) WST-1 solution (200 μL/well; Sigma-Aldrich). Here, a well filed with a WST-1 solution only (without any cells) was used as a blank control. After an incubation time of 1 h, 100 μL of the solution was removed from each well out and transferred into a well of a fresh 96-well plate. The optical densities (OD) of these transferred samples were then obtained at a wavelength of 450 nm using a microplate reader (ABSPlus, Molecular Devices, UK). The cell viability was then calculated based on the following formula 1:
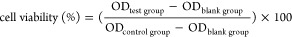
1

### Suture Colonization Tests with Cells

Sterile suture pieces (with the length of 2 cm each) were first incubated in complete cell culture medium for 10 days, and then were transferred to a 12-well plate. Then, HeLa cells or NIH/3T3 cells were seeded into the well plate at the density of 2 × 10^5^ cells/well and coincubated with the sutures. After 24 h, the cell medium was replaced with a Live/Dead staining solution (650 μL/well; Invitrogen, L3224) and the samples were incubated for 20 min at 37 °C; afterward, the sutures were transferred to a fresh 12-well plate filled with Dulbecco’s phosphate-buffered saline (D-PBS; 1.0 mL/well). Cells colonizing the suture surface were then visualized on a DMi8 Leica microscope in both phase contrast and fluorescent mode (excitation/emission wavelength: 495/519 nm and 560/630 nm, respectively) using a 10X objective (N PLAN 10*X*/0.25 DRY). The number of cells per suture piece and the length of the suture piece in each image were measured using a software provided by Leica (LAS X, version: 3.0.4.16529). Finally, the cell density on each suture piece was calculated by dividing the determined cell amount by the length of the suture.

### Inhibition Zone Tests

The antibacterial study was conducted against two bacterial strains, *Staphylococcus aureus* (*S. aureus*) and *Escherichia coli* (*E. coli*). following a previously published protocol^48^ with minor changes. In brief, bacterial suspensions were prepared as follows: a single colony of each bacterial strain (*S. aureus* and *E. coli*) was transferred into separate 15 mL falcon tubes containing 10 mL of Luria–Bertani (LB) medium. The tubes were then incubated in a shaking incubator at 37 °C for 24 h. After incubation, a 50 μL aliquot of each bacterial culture was transferred into a UV-cuvette containing 1.0 mL of fresh LB medium. The OD at 600 nm of each suspension was adjusted to 0.015–0.017 to standardize the bacterial concentration for the experiments. These bacterial suspensions were then used as a stock for subsequent testing. Before the inhibition tests, the suture pieces (2 cm in length each) were washed with 80% ethanol for 30 min and then exposed to UV-light in a tissue culture plate for 1 h. For the inhibition zone test, 20 mL of agar medium was poured into plastic Petri dishes and allowed to solidify. A 25 μL aliquot of the bacterial suspension was spread evenly across the surface of the agar in each Petri dish. The suture samples were then placed onto the agar plates, and a control plate (without any suture samples) was prepared. Each plate was incubated at 37 °C for 24 h. The following day, images of the Petri dishes were captured, and the inhibition zones around each suture sample were measured to quantify the antibacterial effectiveness of each suture piece. The size of the inhibition zones was calculated from the images using ImageJ^49^ (Version 1.54k, National Institutes of Health, Bethesda, Maryland, USA).

### Production of Amine-Modified, Copper-Doped Mesoporous Bioactive Glass Nanoparticles (aCu-MBGNs)

Copper-doped mesoporous bioactive glass nanoparticles (Cu-MBGNs) were synthesized following previously published protocols^50,51^ with slight modifications. First, a Cu/ascorbic acid complex was prepared: 1.71 g of copper(II) chloride dihydrate (CuCl_2_·2H_2_O, purity ≥99.99%, Sigma-Aldrich) was dissolved in 50 mL of D.I water and heated to 80 °C under magnetic stirring until fully dissolved. Then, 50 mL of a 0.4 M aqueous solution of l-ascorbic acid (purity ≥99.0%, Sigma-Aldrich) was added to the copper(II) chloride (CuCl_2_) solution, and the reaction was allowed to proceed for 24 h at 80 °C with continuous stirring. The resulting mixture was centrifuged at 7830 rpm for 15 min, and the supernatant containing the Cu/ascorbic acid complex was stored in a refrigerator for future use. For the synthesis of Cu-MBGNs, mesoporous bioactive glass nanoparticle (MBGN) precursors (70% silicon dioxide (SiO_2_)/30% calcium oxide (CaO); values denote mol %) were prepared using a microemulsion-assisted sol–gel method. In brief, 8 mL of ethyl acetate was dissolved in 26 mL of a 22.0% (w/v) aqueous cetyltrimethylammonium-bromide (CTAB) solution and stirred for 30 min. Then, 5.6 mL of ammonia solution (1.0 M) was added, followed by an additional 15 min of stirring. Next, 2.88 mL of tetraethyl orthosilicate (TEOS) and 1.83 g of calcium nitrate (Ca(NO_3_)_2_) were sequentially added at 30 min of intervals. To incorporate copper, 2 mL of the Cu/ascorbic acid complex was added to the mixture. After 4 h of stirring, the resulting product was centrifuged at 7830 rpm for 20 min, washed three times with D.I water and ethanol, and dried at 60 °C overnight. The dried powders were then calcined at 700 °C for 3 h with a heating rate of 2 °C per min. For amino-functionalization of the particles, a post-treatment was applied following a modified version of the approach described in ref.^52^ Briefly, 200 mg of the powder and 5.0% (v/v) (3-aminopropyl)triethoxysilane (APTES) were added to 20 mL of anhydrous toluene and refluxed with stirring at 80 °C for 6 h. Lastly, the resulting product was washed with toluene by centrifugation and dried at 80 °C for 48 h. Upon amination, the zeta potential of the Cu-MBGNs was increased from ≈ - 22.0 mV to ≈ + 16.0 mV for aCu-MBGNs (Figure S1, Supporting Information).

### Creating aCu-MBGN Coatings on Mucin Coated Sutures

To further functionalize mucin coated sutures with aCu-MBGNs, the sutures were incubated directly after the mucin coating procedure in a 15 mL of 10 mM MES buffer (pH 5.0) containing 20 mg EDC and 20 mg NHS for 30 min. After this incubation step, a 20 mL aqueous solution of aCu-MBGNs (prepared at a concentration of 0.1% (w/v)) was added to the MES solution. After an overnight incubation step, the samples were rinsed with 80% (v/v) ethanol and distilled water and finally allowed to dry at room temperature under a fume hood.

### SEM Images

The surface morphology of the sutures was analyzed by employing scanning electron microscopy (SEM, Auriga CrossBeam, Carl Zeiss Microscopy GmbH, Jena, Germany). Samples were mounted onto aluminum stubs that were covered with conductive carbon tape, and images were acquired at different magnifications using an accelerating voltage of 1.0 kV. The size of the nanoparticles attached to the suture surface was then analyzed by randomly selecting ∼50 particles and measuring their major and minor axis using ImageJ.

### Drug Release

To allow for a controlled release of a drug molecule from a mucin coating layer, a previously published protocol^53^ was adapted with slight changes. In brief, mucin coated and uncoated sutures pieces (∼22.5 cm in length each) were incubated for 5 h in a tetracycline hydrochloride (TCL, AppliChem GmbH, Darmstadt, Germany) solution (2 mg mL^–1^) prepared in UPW. Then, 2 mL of a 90% (v/v) glycerol solution was gradually added while gently shaking the preincubated samples. Next, the condensed mucin layer was stabilized using 1.0 mL of a 60% (v/v) glycerol solution containing cationic agents (0.2% (w/v) poly-l-lysine (PLL) and 600 mM magnesium dichloride (MgCl_2_)). After overnight incubation, the sutures were thoroughly washed with UPW, and 2 mL of 154 mM NaCl was added to open the condensed mucin chains and to release the entrapped TCL. To analyze the TCL release over time, 100 μL of the supernatant was collected at each time point and characterized using a multimode microplate reader (Varioskan LUX, Thermo Fisher Scientific, Vantaa, Finland). Throughout the spectroscopic measurements, the samples were maintained at 37 °C and 40 rpm in an incubator.

### Statistical Analysis

To perform statistical analyses, the software OriginLab (Northampton, Massachusetts, USA) was used. First, a Shapiro–Wilk test was conducted to assess the distribution of the measured values. Then, a two-sample Student’s *t* test was employed for normally distributed populations with similar variances, whereas a two-tailed Welch’s *t* test was used for normally distributed populations with significantly different variances. Additionally, a Mann–Whitney test was applied to non-normally distributed data (*e.g.*, for comparing the TCL release between the “NaCl” and “UPW” groups at 9 h). To detect statistical differences between more than three groups with non-normally distributed populations (*i.e*., for the TCL release determined at 48 h), Kruskal–Wallis ANOVA was performed, followed by Dunn’s tests for multiple comparisons. In all cases, a *p*-value of 0.05 (corresponding to a confidence level of 95%) was used as the threshold for significance; significant differences were denoted with an asterisk (*) and nonsignificant differences with ‘n.s.’. All statistical tests were performed using the sample sizes indicated in the respective figure captions.

## Results and Discussion

**Figure 1:**
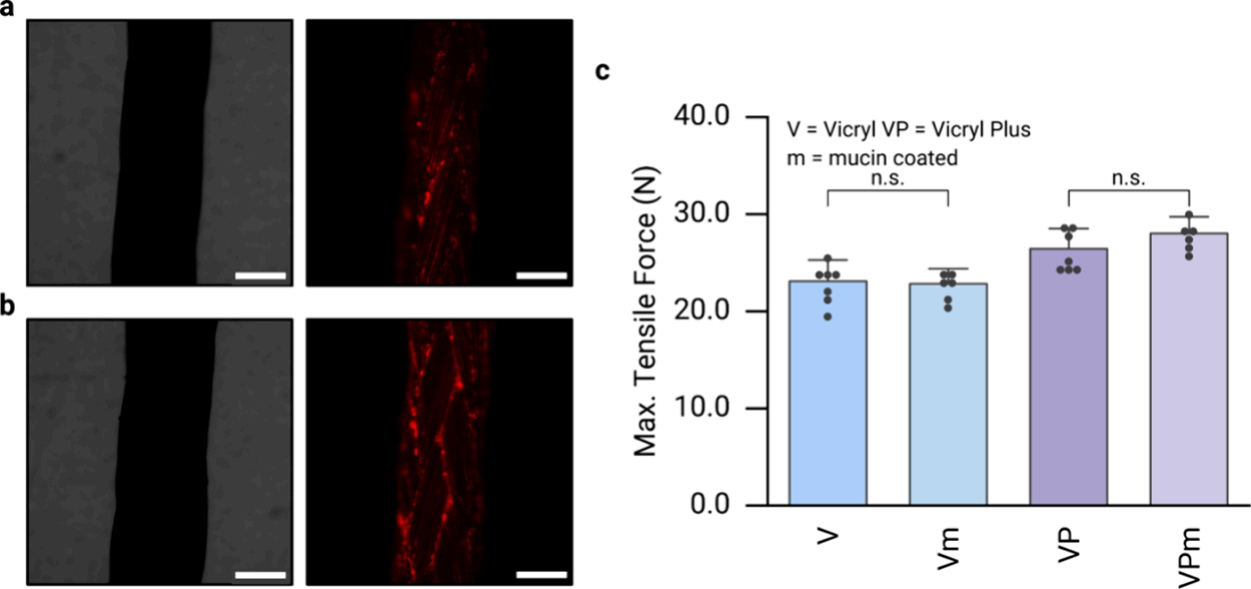
Optical and mechanical characterization of mucin coated sutures. Mucin-coated Vicryl (a) Vicryl Plus (b) samples were imaged on a microscope in bright field mode (left) and in fluorescence mode (right). ATTO-594 labeled mucin was used and the exposure time for capturing the fluorescence images was set to 100 ms. Scale bars represent 250 μm. (c) Maximum tensile force determined for different suture variants: Vicryl (V), Vicryl Plus (VP), and their mucin coated variances Vicry mucin (Vm) and Vicryl Plus mucin (VPm). Data shown represents mean values; error bars denote the standard deviation as calculated from *n* ≥ 6 independent samples. “n.s.” indicates statistically nonsignificant differences (based on a *p*-value of 0.05).

To verify the successful grafting of mucin macromolecules onto commercial Vicryl sutures, we employ light microscopy. From the bright field image of uncoated Vicryl sutures, their diameter can be estimated to be ∼280 μm; and when imaged in the red channel of a fluorescence microscope, these uncoated sutures appear dark, indicating the absence of any autofluorescence in this wavelength range (Figure S2a, Supporting Information). In contrast, sutures coated with ATTO-594 labeled mucin exhibit a bright red fluorescence signal when imaged in the same channel (Figure 1a); at an exposure rate of 100 ms, even the braided structure of the suture can be observed. Very similar results are obtained for Vicryl Plus sutures: uncoated samples are dark when imaged in fluorescence mode (Figure S2b, Supporting Information), whereas a clear red signal visualizes the braided substructure of a mucin-coated sample (Figure 1b). Furthermore, the fluorescence intensities obtained for either coated suture variant are comparable, which suggests that that the presence of triclosan on the surface of the Vicryl Plus variant did not interfere with the mucin coating procedure.

Additionally, the fluorescence signal of the mucin coating on Vicryl (Figure S3a) and Vicryl Plus (Figure S3b) remains detectable after the initial physically attached mucin layer is washed off; here, the signals obtained after 7 days (Figure S3c, d) and 14 days of incubation at 40 rpm and 37 °C (Figure S3e, f) appear similar, indicating stable mucin attachment. In contrast, the fluorescence signal obtained from physically attached coatings on Vicryl (Figure S4a) and Vicryl Plus (Figure S4b) becomes nearly undetectable after 3 days of incubation (Figure S4c, d) under the same conditions.

Next, we assess whether the employed coating procedure maintains the mechanical stability of the sutures. To evaluate this aspect, we measure the maximum tensile force of uncoated sutures, which will serve as a baseline value. The average rupture force values for Vicryl and Vicryl Plus samples are determined to be ≈23 N and ≈26 N, respectively (Figure 1c). For mucin coated samples, these average rupture force values are very similar as we measure ≈23 N for Vicryl and ≈28 N for Vicryl Plus samples (Figure 1c). These results suggest that the coating process does not negatively affect the tensile strength of either suture variant.

**Figure 2:**
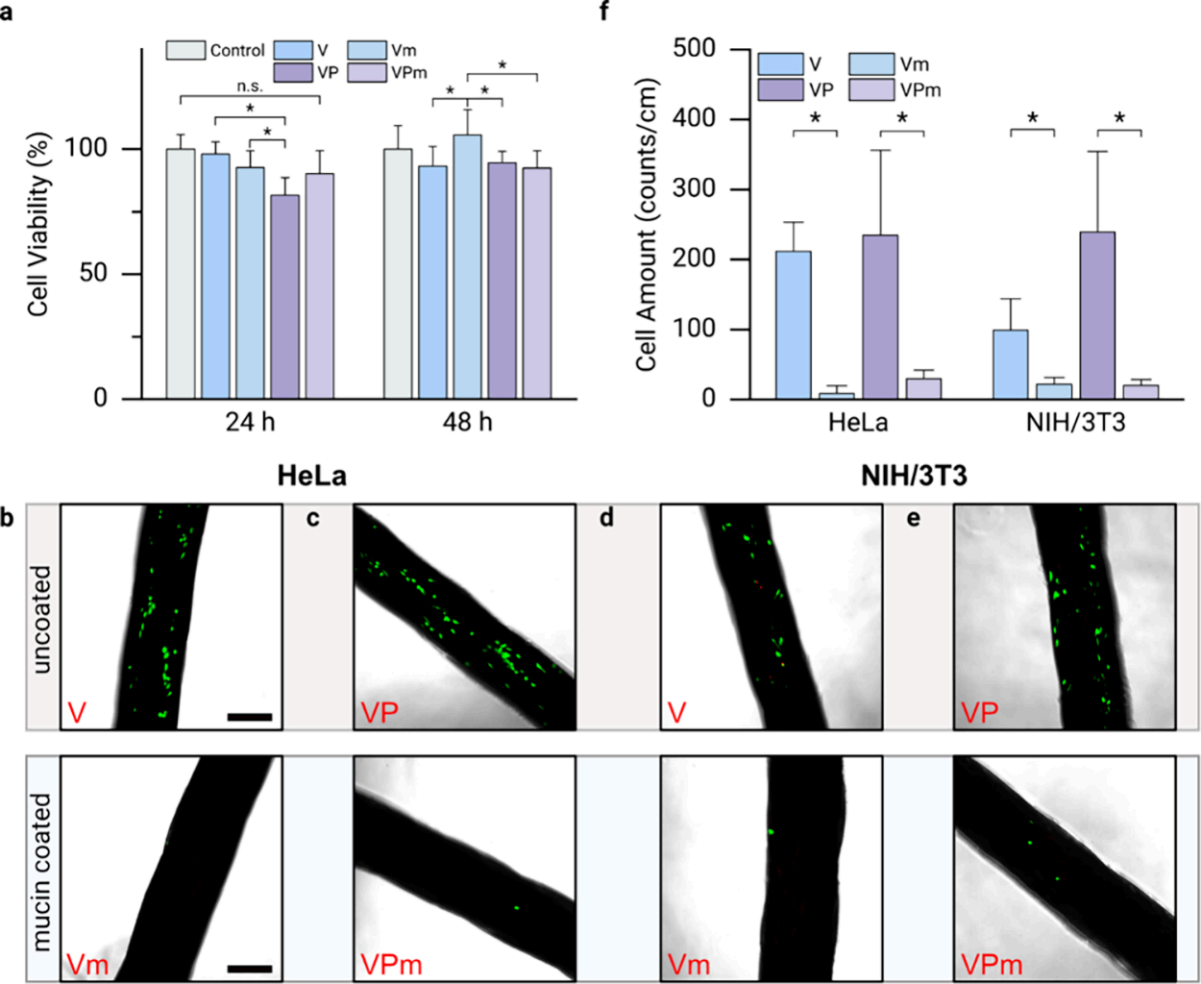
Interaction of different suture variants (Vicryl (V), Vicryl Plus (VP), and their mucin-coated counterparts Vm and VPm, respectively) with eukaryotic cells*.* (a) Viability of HeLa cells after conducting an cytotoxicity test with the sutures. Data shown represents mean values; error bars denote the standard deviation as calculated from *n* = 6 independent samples. (b–f) Suture colonization tests conducted with HeLa and NIH/3T3 cells. The exemplary fluorescence microscopy images (b–e) show live (green) and dead (red) HeLa and NIH/3TC cells, respectively. The scale bar in (b) denotes 250 μm and applies to all images of this figure. Data shown in (f) represents mean values determined from such microscopy images; error bars denote the standard deviation as calculated from *n* = 4 independent samples. Asterisks and “n.s.” indicate statistically significant and nonsignificant differences, respectively (based on a *p*-value of 0.05).

Having compared the tensile properties of uncoated and mucin-coated sutures, we now evaluate their biocompatibility by conducting an cytotoxicity assay; here, the response of HeLa cells to a leaching medium generated from the different suture variants (*i.e*., a cell medium that was incubated with a particular suture variant) is assessed at incubation times of 24 and 48 h. Overall, we find that the cell viability remains high as none of the obtained viability values falls below the minimum accepted threshold of ≈70%.^54^ This result indicates a robust biocompatibility of the materials used here and suggests that any potential residues left on the sutures from the coating process are unproblematic. Nevertheless, after 24 h, we observe a slight but significant decrease in the cell viability when comparing the “uncoated Vicryl Plus” group to the “uncoated Vicryl” group. This difference implies that a release of triclosan from the Vicryl Plus sutures might affect the cell viability; and indeed, triclosan has been reported to be cytotoxic for several cell lines.^55,56^ Interestingly, this reduction in viability is not observed for Vicryl sutures carrying a mucin coating; moreover, the negative effect brought about by the triclosan is less pronounced when a mucin coating is applied to the Vicryl Plus sutures: now, we find a high level of viability that is comparable to that of the control group (Figure 2a). At 48 h of incubation time, the viability of all cell groups is either comparable or even somewhat larger than at the shorter incubation time. Notably, the mucin-coated Vicryl group exhibits a significantly higher cytocompatibility than the three other suture variants.

Furthermore, based on previous findings obtained with mucin coatings, we anticipate that the mucin coated sutures should efficiently be shielded from excessive cell colonization,^57,58^ and this could help reducing inflammatory responses.^59,60^ To test our expectation, the sutures are first immersed into cell culture medium for 10 days to allow for the formation of a conditioning film driven by the unspecific adsorption of proteins; afterward, the ability of eukaryotic cells to colonize the sutures is assessed. As expected, the mucin coating strongly reduces the surface colonization of either suture variant by both, epithelial cells (HeLa) (Figure 2b, 2c) and fibroblasts (NIH/3T3, Figure 2d, 2e): For HeLa cells, we find that the mucin coating reduces the number of attached cells (counts cm^–1^) by more than 90%, and the attachment of NIH/3T3 cells is reduced by more than 80% (Figure 2f). Importantly, as we used a live/dead staining kit to label the cells for microscopy analysis, we can visually confirm our findings from the cytotoxicity test: we do not find any dead cells on the mucin coated sutures, which underscores their excellent biocompatibility.

**Figure 3:**
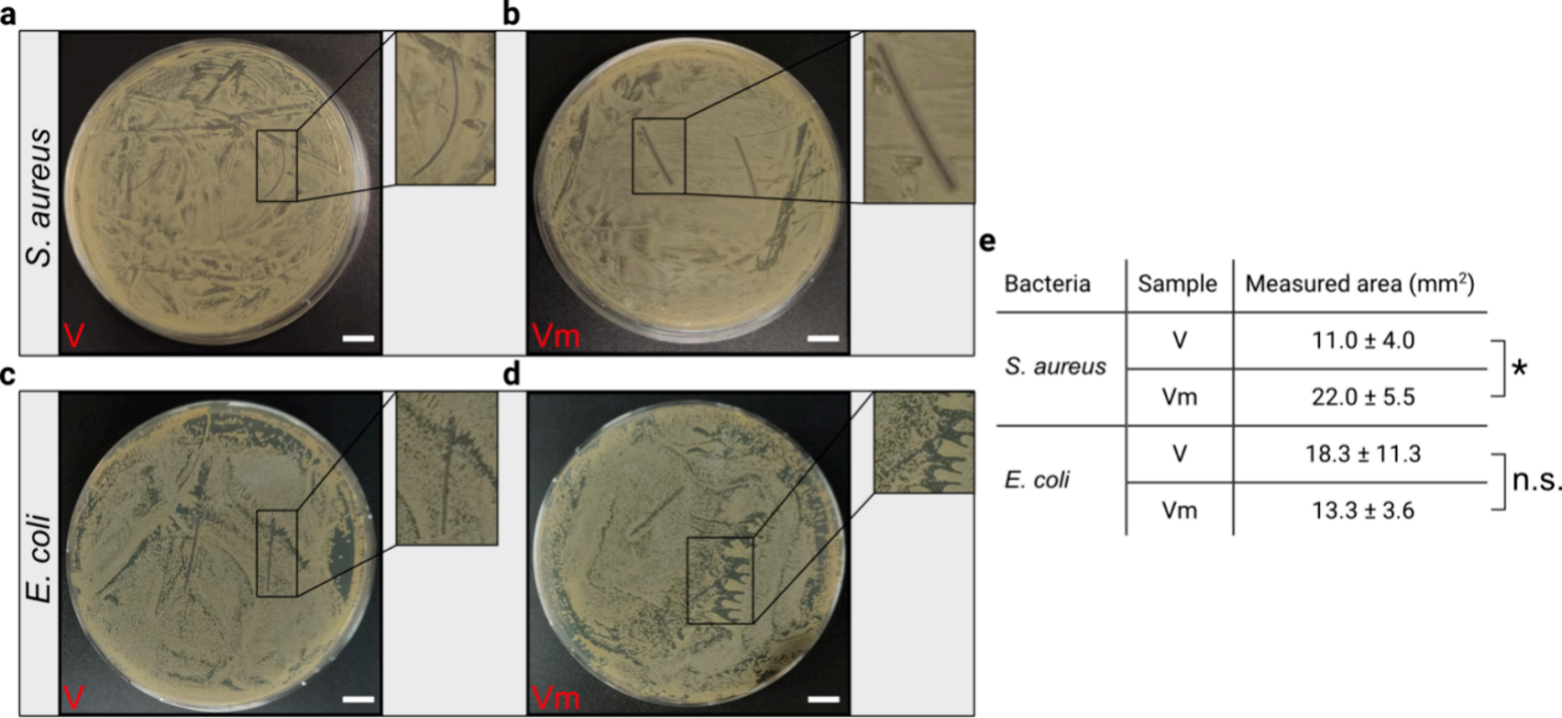
Antibacterial properties of different suture variants. Exemplary agar plate images showing the inhibition zones created by uncoated Vicryl sutures (V) and mucin-coated Vicryl (Vm) sutures toward *S. aureus* (a, b) and *E. coli* (c, d). Scale bars represent 10 mm. (e) Quantification of the inhibition zones created by suture pieces of 2 cm length. Data shown in the table represents mean values together with the standard deviation calculated from *n* = 4 independent samples. Asterisks and “n.s.” indicate statistically significant and nonsignificant differences, respectively (based on a *p*-value of 0.05).

In addition to repelling eukaryotic cells, previous results suggest that a mucin coating can also provide moderate antibiofouling properties toward bacterial colonization.^57,61^ To verify this notion, we conduct tests with the Gram-positive bacterium *S. aureus*, which is capable of rapidly developing antibiotic resistance and is the most common cause of SSIs.^62,63^ When we examine the results of inhibition zone tests conducted with this bacterium, we note that uncoated Vicryl sutures provide virtually no defense against this strain (Figure 3a). In contrast, a clear inhibition zone is formed around mucin-coated Vicryl sutures (Figure 3b), which is significantly larger than the one obtained for uncoated Vicryl sutures (Figure 3e). When assessing the inhibition zones obtained in similar tests conducted with the Gram-negative bacterium *E. coli*, we observe a thin inhibition zone for both, uncoated and mucin-coated sutures (Figures 3c, 3d); however, we do not detect a significant difference between the size of the two zones (Figures 3e).

**Figure 4:**
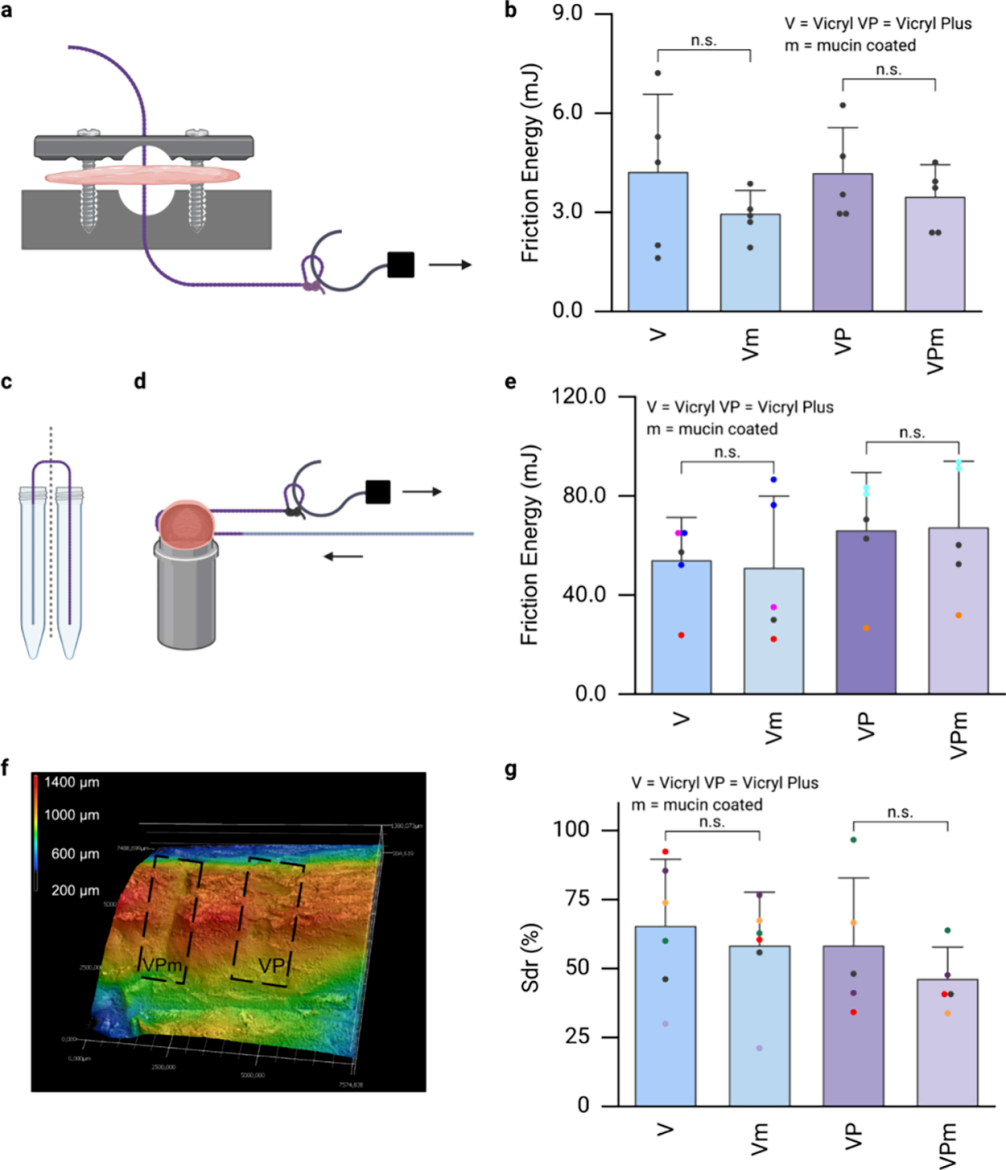
Friction and wear tests performed with different suture variants*.* (a) A first set of friction tests was conducted on perforated porcine skin samples, and the corresponding friction energy values are shown in (b). (c) By employing a modified suture coating procedure (in which only half of each suture is coated), the same chicken stomach sample could be used for up to four consecutive friction tests (d). The corresponding friction energy values are shown in (e); here, identical colors represent data obtained from the same suture/tissue combination. (f) Exemplary profilometry image depicting wear scars on a chicken stomach sample as inflicted by the sliding process of a mucin coated Vicryl Plus (VPm) suture and its uncoated counterpart (VP), respectively. (g) *S*_dr_ values were calculated from profilometric images to compare the local surface roughness of the damaged tissue samples after the sliding tests. Data points obtained on the same tissue sample are represented by identical colors. In all graphs, the data shown represents mean values, and error bars depict the standard deviation as calculated from *n* ≥ 5 independent samples. “n.s.” indicates statistically nonsignificant differences based on a *p*-value of 0.05. The schematic drawings were created using BioRender: https://BioRender.com/c83j150.

Having demonstrated that the mucin coating provides the sutures with strong cell-repellent properties and antibacterial properties, we proceed to evaluate another mechanical parameter that is crucial for the clinical performance of the sutures: their friction behavior. The as-received sutures are already coated with a lubricating layer of calcium stearate.^44^ To test if the mucin coating enhances or reduces the lubricity brought about by this stearate layer, we perform friction tests. In detail, we conduct *ex vivo* friction experiments with animal tissue samples and compare uncoated sutures to their mucin-coated counterparts. For a first set of tests, we utilize porcine skin with circular perforations that are slightly smaller in diameter than the sutures. The sutures are then threaded through these perforations and pulled through the skin sample over a net distance of ∼9 cm (Figure 4a). The corresponding average friction energies obtained for uncoated Vicryl and Vicryl Plus sutures are both determined to be ≈4.2 mJ (Figure 4b). Interestingly, after the application of the mucin coating, the average friction energy values for Vicryl and Vicryl Plus sutures are slightly decreased to ≈3.0 mJ and ≈3.5 mJ, respectively (Figure 4b). Although these changes are not statistically significant, the slight reduction in friction energy might be attributable to the lubrication properties of mucin coatings reported elsewhere^42,47^ – albeit weaker than reported previously. However, the latter is not surprising since the friction tests are here performed on semidry porcine skin samples, and this is not ideal for evoking hydration lubrication,^64^ one of the key mechanisms behind the lubricity brought about by mucin coatings. A second set of friction tests is conducted with chicken stomachs and is supposed to test the sliding behavior of the sutures at a higher tribological load as a given suture is threaded twice through a tissue sample in the geometry of a U–turn. To minimize the impact of sample-to-sample variability arising from using different stomachs, we first perform multiple pulling experiments on the same chicken stomach sample. Here, we verify that the tissue integrity is maintained after conducting three consecutive pulls of ∼9 cm each, and we confirm that differences between the distinct friction profiles obtained during those three consecutive tests are negligible (see Figure S5, Supporting Information). Then, we test long sutures threads where only one-half of the suture is coated (Figure 4c). With those semicoated sutures, we conduct consecutive friction tests using the same stomach sample for a given suture (Figure 4d); this allows us to observe the transition in the friction response between coated and uncoated suture segments. Those modified friction tests demonstrate that the average friction energies obtained in this high-load scenario are very similar for coated and uncoated suture parts, with values of ≈53 mJ and ≈50 mJ, respectively (Figure 4e). Similarly, Vicryl Plus samples return values of ≈60 mJ in this setup configuration (Figure 4e), with no significant difference between uncoated and coated suture segments. However, we do note that changes in the friction energies occur when transitioning from a coated to an uncoated segment (or *vice versa*); yet these variations are not consistent in direction and thus might be a result of variations in the coating quality achieved with the coating process.

To further analyze the tribological properties of the mucin coating, we performed wear tests to assess the surface damage caused by the sliding of either uncoated or coated sutures across tissue samples. In these wear tests, the sutures were dragged across a tissue surface in a controlled and reproducible manner (see Methods), and the resulting damaged areas were analyzed in terms of their surface roughness using laser scanning profilometry. From the obtained topographical images obtained (Figure 4f), we calculate developed interfacial area ratio (S*_dr_*) values after the sliding tests. When we compare results for mucin-coated sutures to those obtained with their uncoated counterparts (Figure 4g), we find slightly lower values for both suture types. However, those differences are not significant. In other words, the results discussed so far demonstrate that, in comparison to the commercially used stearate coating, a mucin coating provides the sutures with (weak) antibacterial properties without incorporating a bactericidal chemical, it very efficiently prevents the colonization of the suture surface by eukaryotic cells, and it maintains the mechanical stability and good sliding properties of the sutures.

**Figure 5:**
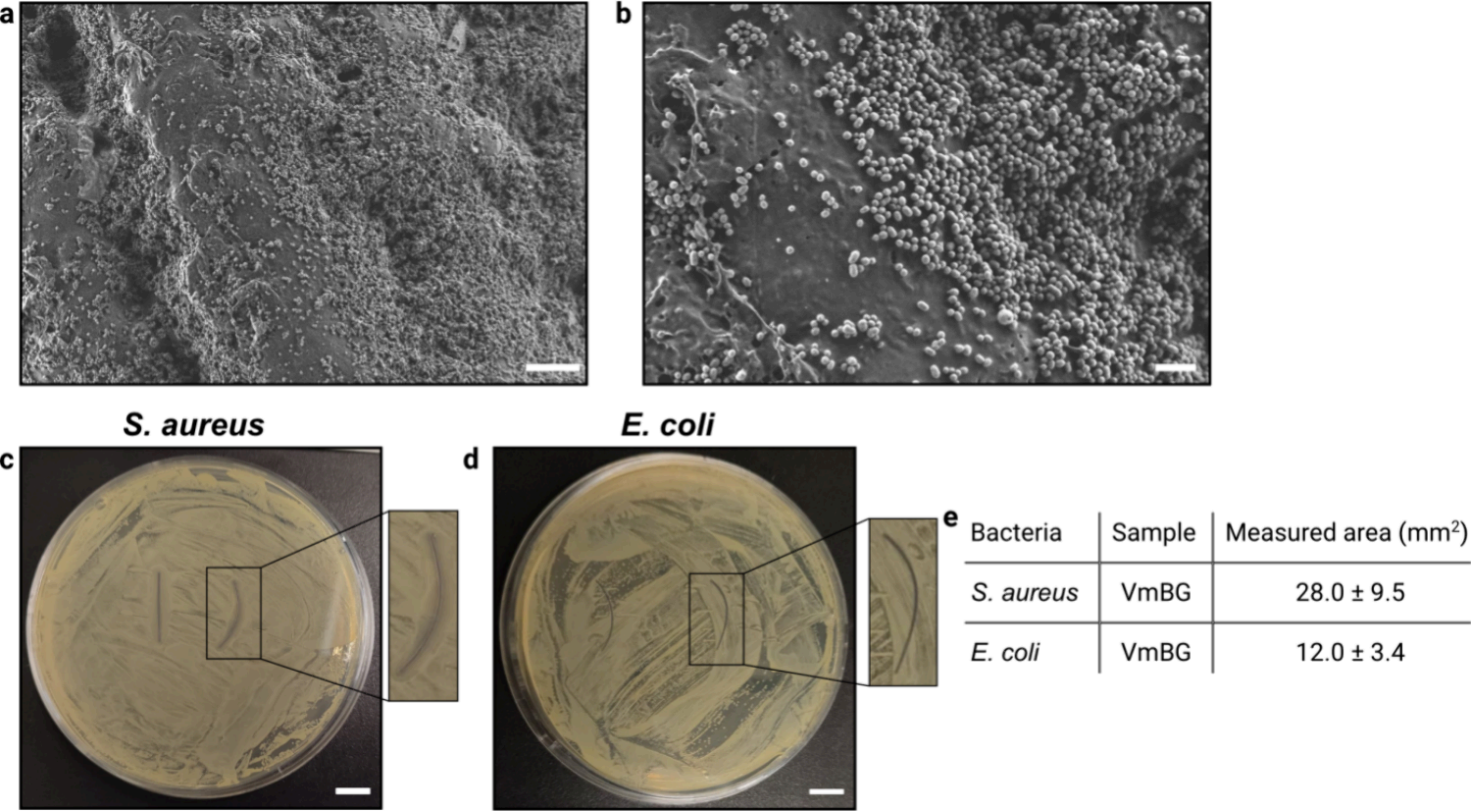
Additional suture modification using aCu-MBGNs. (a, b) SEM images of mucin coated Vicryl sutures carrying aCu-MBGNs (VmBG); the scale bars represent (a) 4 and (b) 1 μm, respectively. Exemplary agar plate images showing the inhibition zones created by VmBG sutures toward *S. aureus* (c) and *E. coli* (d). Scale bars represent 10 mm. (e) Quantification of the inhibition zones created by suture pieces of 2 cm length. Data shown in the table represents mean values together with the standard deviation calculated from *n* ≥ 3 independent samples.

In the last section of this study, we now aim at extending the potential application areas of the sutures to hard tissues, where healing of hard tissues is required, *e.g*., after tooth surgery. To achieve this, we add a layer of amine-modified, copper-doped mesoporous bioactive glass nanoparticles (aCu-MBGNs) onto the mucin coating by employing another cycle of carbodiimide-chemistry (see Methods). In the context of tooth surgery, the bioactive glass nanoparticles could potentially form a thin layer of hydroxyapatite, which would facilitate better bonding to both, bone and soft tissue as well as promote local mineralization as needed.^65,66^ Moreover, when such a bioactive glass coating is applied to the Vicryl sutures, the buffering capacity of this coating can be expected to prolong the integrity and strength of the sutures.^67^ In addition, doping the bioactive glass NPs with biologically active ions represents an interesting approach to enhance the coating antibacterial properties,^68^ as also discussed above. SEM images of such modified Vicryl sutures (VmBG) demonstrate a high density and reasonably homogeneous distribution of bioactive glass nanoparticles across the suture surface (Figure 5a). The nanoparticles appear to be uniform in size and predominantly arranged in a monolayer; they exhibit either spherical or elliptical shapes, with an average major axis of ∼250 nm and an average minor axis of ∼180 nm (Figure 5b, Figure S6, Supporting Information).

Moreover, the aCu-MBGNs on the surface are expected to remain stable under in vitro conditions and to form hydroxyapatite after a certain period of time. Notably, during incubation in simulated body fluid (SBF) at 37 °C for 14 days, the bioactive glass distribution on the surface of the VmBG sample remains stable (Figure S7), and cauliflower-like structures are observed, indicating the initial steps of hydroxyapatite formation.^69^ As the bioactive glass NPs are doped with copper ions, this might enhance the antibacterial properties of the mucin coating; and indeed, for tests conducted with *S. aureus*, we find larger inhibition zones for VmBG sutures (*i.e*., ≈ 28 mm^2^, see Figure 5c, e) than for sutures carrying a mucin coating only (Figure 3b, e). For inhibition zone tests conducted with *E. coli*, we do not detect an improvement (Figure 5d, e and Figure 3d, e). This result can be explained by the fact that *S. aureus* is more sensitive toward copper ions than *E. coli*.^70^ In future work, it might be possible to further modify the aCu-MBGNs so that the required time to release the ions from the NPs is reduced or that the oxidation state of copper present in and/or around the mesoporous of the bioactive glass is optimized for improved antibacterial efficiency.^71,72^ Additionally, doping with other nanoparticles, such as silver^73^ to enhance the antibacterial activity or titanium dioxide^74^ to improve the cytocompatibility,^75^ could be a viable alternative.

**Figure 6:**
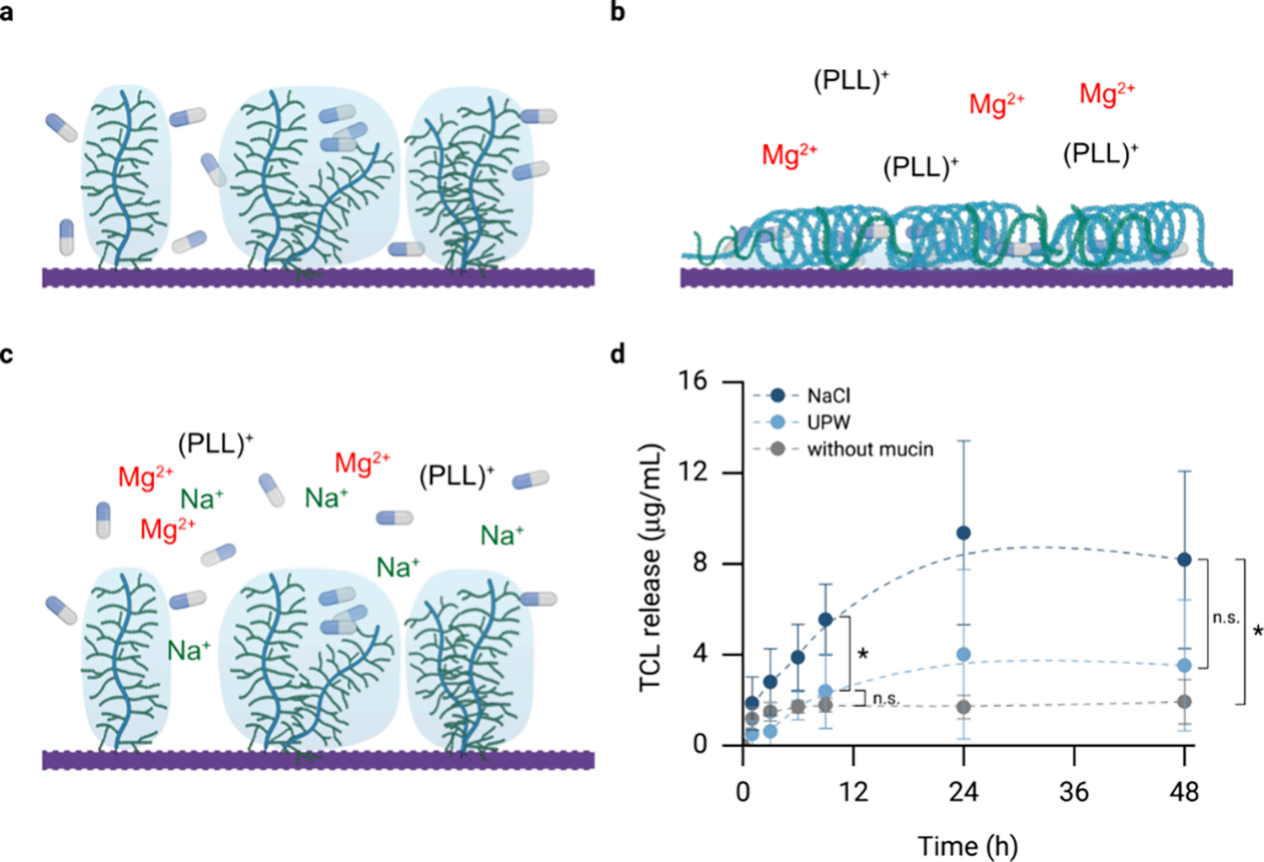
Triggerable drug release from condensed mucin coatings on Vicryl sutures: (a–c) Schematic representation of the process used to (a) load the mucin surface with the target drug TCL; (b) trap the drug through mucin layer condensation and stabilization with cations; (c) release TCL upon contact of the coating with a physiological NaCl concentration. (d) TCL release profiles from uncoated and mucin coated Vicryl sutures obtained in a 154 mM NaCl solution (dark blue symbols) and in UPW (light blue symbols). Data shown in gray represents the control group (uncoated Vicryl sutures). The concentration of released TCL is calculated with the help of a standard curve (Figure S8, Supporting Information). Data shown represents mean values; error bars denote the standard deviation as calculated from *n* ≥ 4 independent samples. Asterisks and “n.s.” indicate statistically significant and nonsignificant differences, respectively (based on a *p*-value of 0.05). The schematic drawings were created using BioRender: https://BioRender.com/v75v402.

Finally, another advantage of the mucin coating introduced here in comparison to the commercial calcium salt coating already present on the Vicryl sutures is that the macromolecular conformation of the mucin coating can be altered, *i.e*., it can be switched from an elongated state into a condensed state (by exposing the coating to glycerol) and vice versa. Here, the condensed state can be transiently stabilized with divalent cations such as Ca^2+^/Mg^2+^ or cationic molecules, and those stabilizing cations can be displaced again by Na^+^ ions.^53^ By leveraging this triggerable conformational change, a drug can be encapsulated into the mucin coating (Figure 6a, b) and then is released upon exposure of the coating to a physiological NaCl concentration (*i.e*., upon contact with human tissue, Figure 6c).

Here, we use this procedure to load a model drug (the antibiotic tetracycline hydrochloride, TCL) into the mucin coating. When such TCL-loaded Vicryl sutures are incubated in either a physiological salt solution (154 mM NaCl) or in UPW, we observe a significantly higher average release of TCL after 8 h in the NaCl environment than in UPW (Figure 6d; however, at longer incubation times of 2 days, this difference is not significant anymore). In contrast, Vicryl sutures that do not carry a mucin coating show no drug release behavior as they cannot be easily loaded with an additional drug after their production.

## Conclusions

In summary, we here describe a highly cytocompatible coating, in which the glycoprotein mucin is covalently attached onto commercially available sutures. Owing to the innate antibacterial properties of mucins, this coating may be considered as a substitute for commercial biocides such as triclosan, which is currently used to convey antibacterial properties to sutures. In addition, the mucin coating efficiently inhibits eukaryotic cell attachment without compromising the stability or tribological properties of the sutures. Moreover, with the mucin coating present on the suture surface, it is possible to utilize the entrapment capability of the mucin layer to enable a localized, triggered drug release. The functional groups present on the mucin glycoprotein make it easy to further modify the sutures, *e.g.*, by adding a layer of aCu-MBGNs to expand the potential application range of the sutures to the treatment of hard tissues. To extend the functionality of the biopolymeric suture coating described here even more, incorporating photosensitizers into the mucin structure that fluorescence when exposed to light could provide an interesting option for monitoring wound healing^76^ (*e.g*., inside the oral cavity) thus offering a highly multifunctional, sustainable, and environmentally friendly coating alternative to advance the field of wound healing.

## Supporting Information Available

Zeta potential of Cu-MBGNs before and after amination, microscopy images of sutures, optical characterization of chemically and physically mucin-coated sutures, consecutive friction measurements, size distribution of aCu-MBGNs, SEM image of VmBG after 14 days of SBF incubation, and standard curve of TCL (PDF)
