# Supplementary material for: Mucin Coatings Establish Multifunctional Properties on Commercial Sutures
Source: ACS Appl Bio Mater. 2025 Feb 27;8(3):2263–74. doi: 10.1021/acsabm.4c01793 (PMC11921033; doi:10.1021/acsabm.4c01793)
Supplement: Supplementary file 1 — mt4c01793_si_001.pdf [file mt4c01793_si_001.pdf]

Supporting Information

Mucin Coatings Establish Multifunctional Properties on Commercial Sutures

Ufuk Gürer[1, 2], Di Fan[1, 2], Zhiyan Xu[3], Qaisar Nawaz[3], Jorrit Baartman[1, 2], Aldo R. Boccaccini[3] , Oliver Lieleg[1, 2] *

[1] Department of Materials Engineering, School of Engineering and Design, Technical University of Munich, Boltzmannstraße 15, 85748 Garching, Germany

[2] Center for Protein Assemblies (CPA), Munich Institute of Biomedical Engineering (MIBE), Technical University of Munich, Ernst-Otto-Fischer Straße 8, 85748 Garching, Germany

[3] Institute of Biomaterials, Department of Materials Science and Engineering, University of Erlangen-Nuremberg, Cauerstraße 6, 91058 Erlangen, Germany

*****corresponding author

Prof. Dr. Oliver Lieleg
Department of Materials Engineering, School of Engineering and Design,
Technical University of Munich,
Boltzmannstraße 11, 85748 Garching, Germany

E-Mail: oliver.lieleg@tum.de,
Phone: +49 89 289 10952

keywords: biopolymer, antibacterial, surgical site infection, bioactive glass, drug release

ORCID IDs: **UG** (0009-0004-0016-7406); **DF** (0000-0001-7291-2592); **ZX** (0009-0000-9854-5364); **QN** (0000-0001-7068-2383); **JB** (0009-0005-7990-6386); **ARB** (0000-0002-7377-2955); **OL** (0000-0002-6874-7456)

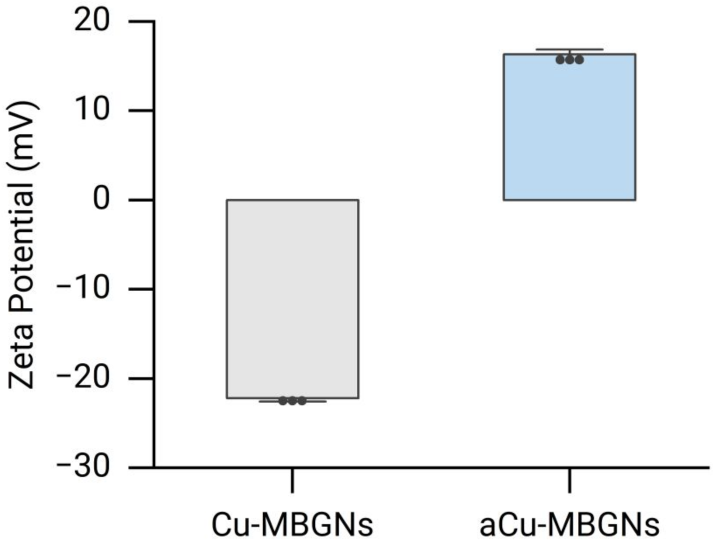

Figure S1. Zeta potentials of Cu-MBGNs before and after amination. Data shown represents average values and standard deviations as calculated from *n* = 3 measurements. The zeta potentials of the particles were measured (Malvern Instruments, UK) in DI water at a concentration of 1.0 mg mL^{-1}.

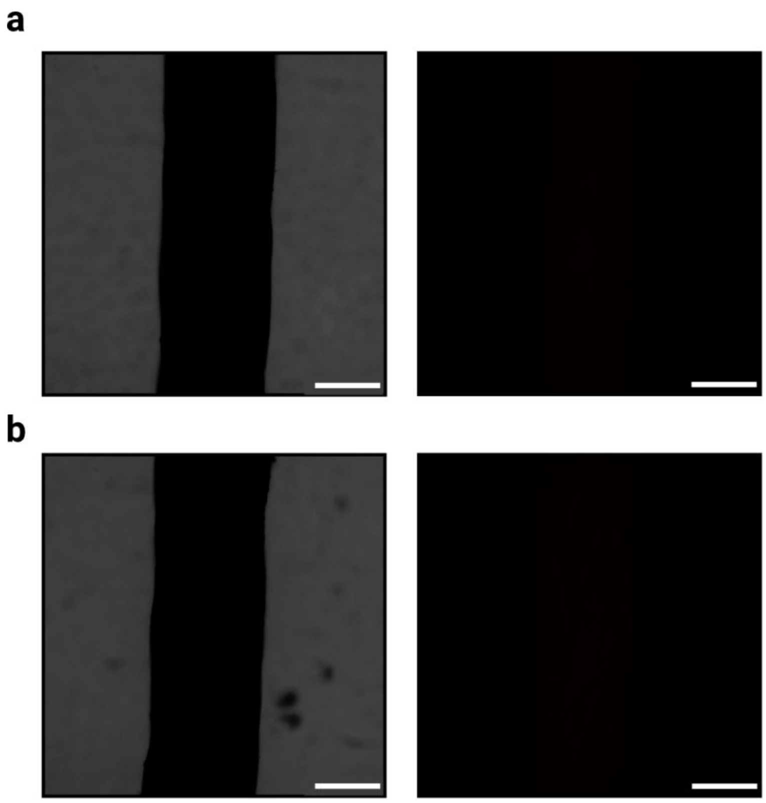

Figure S2. Microscopy images of a) Vicryl and b) Vicryl plus sutures acquired in bright field mode (left) and in fluorescence mode (red channel, right) using an exposure time of 100 ms. The scale bars represent 250 µm.

Stability Test: To investigate the stability of mucin coatings, mucin macromolecules were physically attached to the sutures by incubating the samples during the carbodiimide coupling reaction without adding EDC/NHS. In the other set, the standard coating procedure described in the Materials and Methods section was followed. After overnight incubation in a mucin solution, the samples were rinsed multiple times with 80 % ethanol and D.I water, followed by a brief vortexing step. Bright-field and fluorescent images were captured to characterize the generated coatings. Subsequently, the samples were incubated at 37 °C and 40 rpm for 3, 7 and 14 days, after which additional images were acquired to evaluate the stability of the coatings.

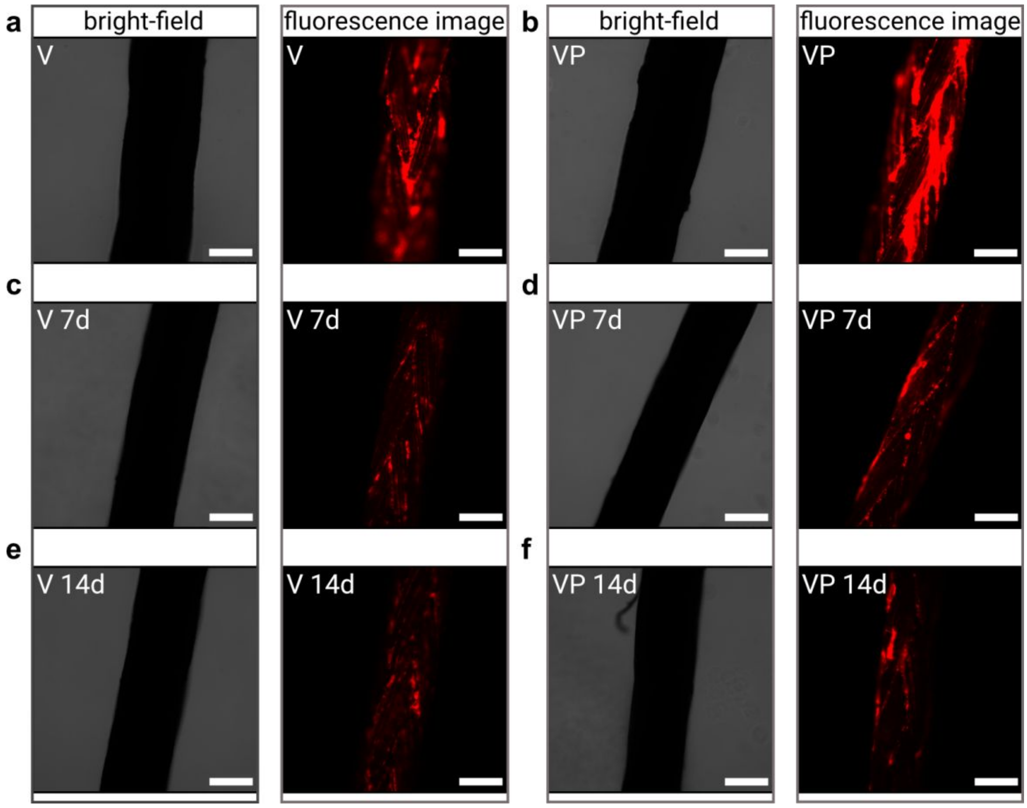

Figure S3. Optical characterization of mucin-coated sutures generated by carbodiimide chemistry: Images of Vicryl (a, c, e) and Vicryl Plus (b, d, f) obtained after the first cleaning step (top row), after 7 days of incubation (middle row) and after 14 days of incubation (bottom row). All samples were imaged using a microscope in bright-field mode (left) and fluorescence mode (right). ATTO-550-labeled mucin was used to generate the coatings; the exposure time for imaging was set to 400 ms. Scale bars represent 250 µm.

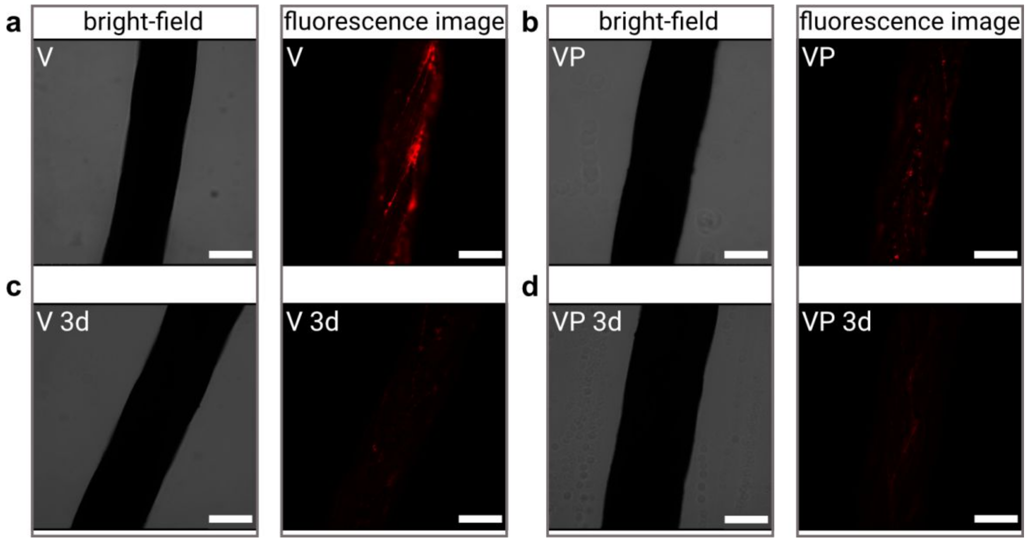

Figure S4. Optical characterization of mucin-coated sutures generated by passive mucin adsorption. Images of Vicryl (a, c) and Vicryl Plus (b, d) obtained after the first cleaning step (top row) and after 3 days of incubation (bottom row). All samples were imaged using a microscope in bright-field mode (left) and fluorescence mode (right). ATTO-550-labeled mucin was used to generate the coatings; the exposure time for imaging was set to 400 ms. Scale bars represent 250 µm.

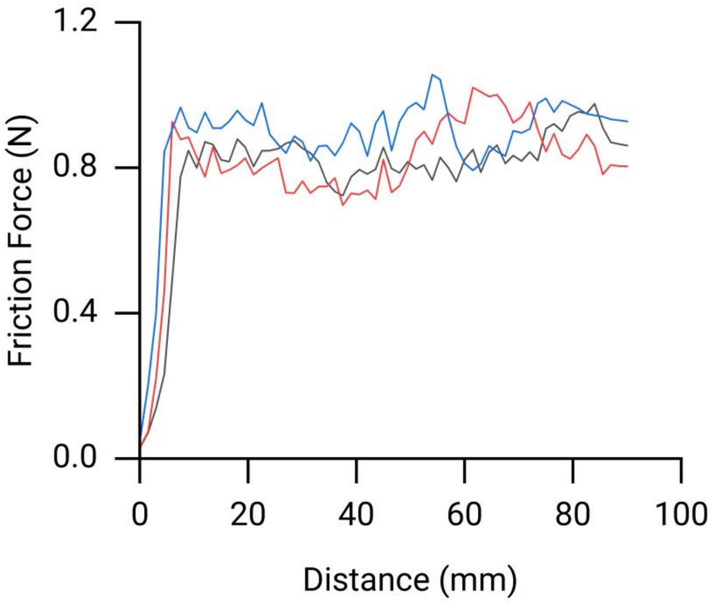

Figure S5. Friction versus distance data from three consecutive measurements (using a suture of 45 cm in length) were conducted with the very same chicken stomach sample. Each colored curve represents a ~ 9 cm pull of the suture through the tissue. The highly similar force profiles and force values across the three pulls indicate that the tissue remains intact and unaffected by the repeated pulling process.

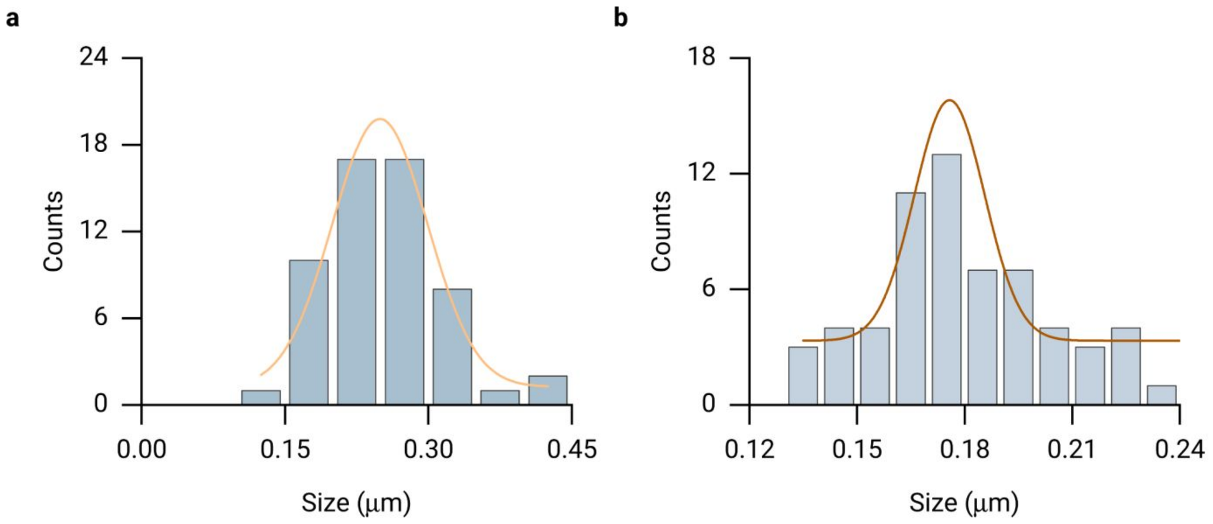

Figure S6. Size distribution of the a) major and b) minor axes of amine-modified, copper-doped mesoporous bioactive glass nanoparticles (aCu-MBGNs) as measured from SEM images using ImageJ (*n* ≥ 50 particles). The curves represent Gaussian fits to the distributions, from whose peak positions the average dimension in each direction was determined.

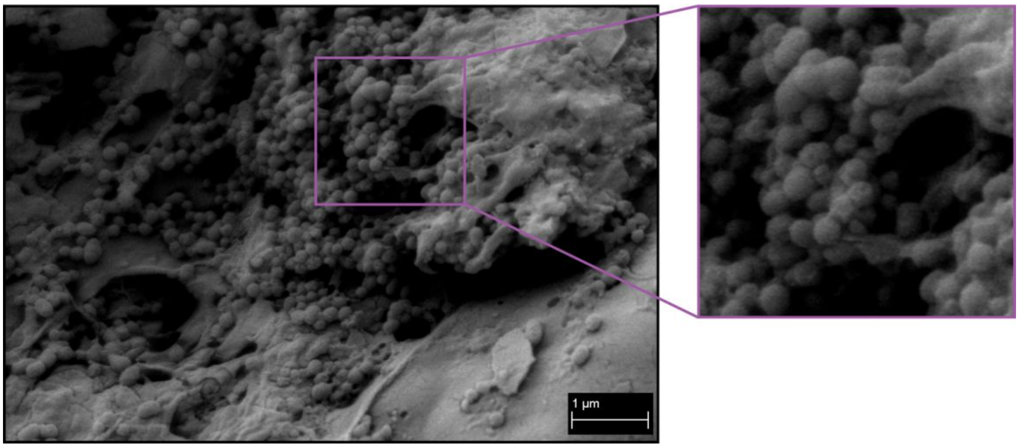

Figure S7. SEM image (25.0 KX magnification) of mucin-coated Vicryl suture carrying aCu-MBGNs (VmBG) after 14 days of incubation in simulated body fluid (SBF).

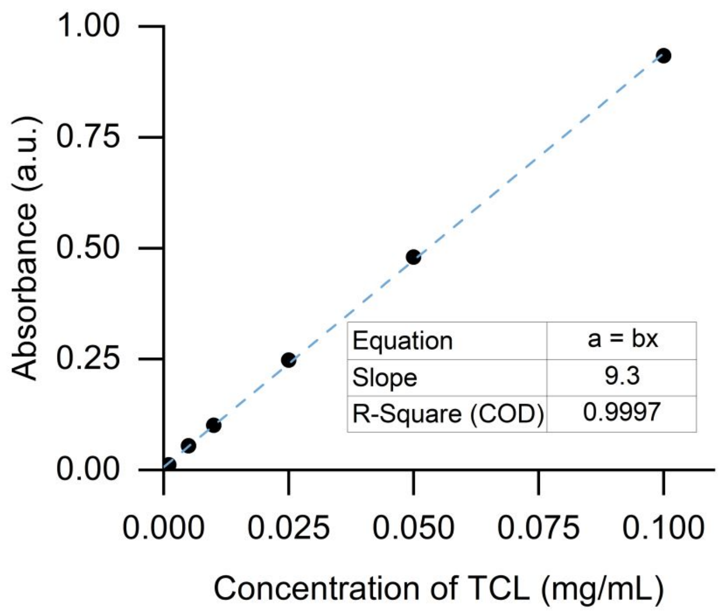

Figure S8. Standard curve for tetracycline hydrochloride (TCL) illustrating the correlation between TCL concentration and absorbance. A linear fit (dashed blue line) indicates the slope. Each data point represents the average of $n = 3$ samples.
